# Sex matters in Alzheimer’s disease?

**DOI:** 10.18632/aging.203950

**Published:** 2022-03-12

**Authors:** Sheeja Navakkode, Toh Hean Ch’ng, Sreedharan Sajikumar

**Affiliations:** 1 Lee Kong Chian School of Medicine, Nanyang Technological University, Singapore 308232; 2 School of Biological Sciences, Nanyang Technological University, Singapore 637551; 3 Department of Physiology, National University of Singapore, Singapore 117597; 4 Life Sciences Institute Neurobiology Programme, National University of Singapore, Singapore 117456; 5 Healthy Longevity Translational Research Programme, Yong Loo Lin School of Medicine, National University of Singapore, Singapore 117456

**Keywords:** Alzheimer's disease, LTP, behavioural tagging, sexual dimorphism, transcriptome profiling

Alzheimer’s disease (AD) is the most common cause of dementia amongst the aging population. According to the World Health Organization (WHO), currently there are more than 55 million people (8.1% of women and 5.4% of men over 65 years) living with dementia. Globally, there are more women with AD than men and this pattern is expected to continue. Therefore, studies looking at the underlying mechanisms of sex differences in AD may be instrumental in developing custom-tailored strategies for early detection, prevention and treatment of AD.

Synaptic dysfunction is an early event in AD and a good predictor of cognitive deficits [1,2]. How sex differences and synaptic function impacts AD pathology, leading to disruption in neuronal connectivity remains an active area of research [3]. One approach to study this phenomenon is to examine how synaptic strengthening initiated by various synaptic activity differs between males and female AD mice in the hippocampal Schaefer-collateral pathway.

In our study, we demonstrated that APP/PS1 female mice show a faster decline in long-term potentiation (LTP) – which is a cellular correlate of learning and memory- as compared to male mice [4,5]. We compared the responses in males and females brain slices using three different stimulation paradigms: strong tetanic stimulation, theta burst stimulation and spike- timing dependent plasticity to induce LTP [5]. In all three cases, the faster decline in LTP in females is comparable and robust, yet distinct from male mice. These brain slice studies are further complemented with behavioral tagging (BT) experiments looking at formation of associative memories in AD mice. In these rodent memory tasks, female AD mice performed more poorly than their male counterparts in formation and recall of long-term memories. BT paradigm was employed to study differences in associative memory as it can reveal subtle behavioural differences that are normally masked in standard memory tasks [6].

To characterize molecular changes in the AD brain that is attributed to sex differences, we performed RNA sequencing and immunohistochemistry of the hippocampus and showed accelerated pathology, stronger immune response and higher microglial activation in AD female mice compared to males [5]. Strikingly, we noted that plasticity-related genes are more strongly downregulated in females than in males. Finally, we also identified several sex-regulated differentially expressed genes in AD mice. Future studies will be focused on dissecting local signalling cascades responsible for the observed differences in synaptic deficits as well as finding targets to reverse the impaired plasticity in various AD models.

**Figure 1:**
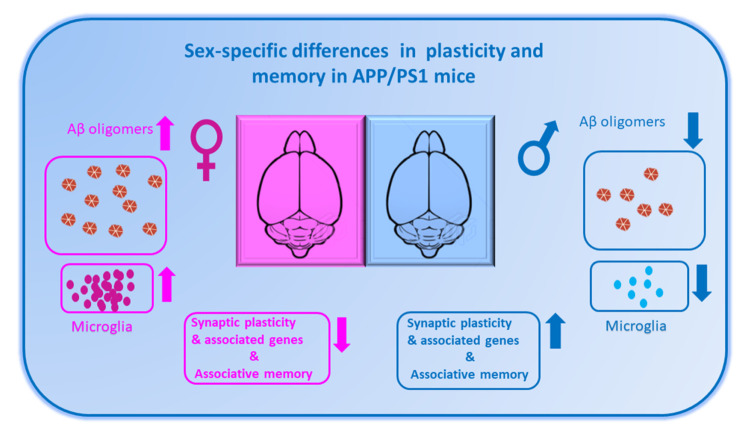
The diagram shows sex-specific alterations in plasticity and memory and the associated changes in amyloid beta (Aβ) pathology and inflammatory response in APP/PS1 mice. Female AD mice (represented as pink) shows a faster decline in synaptic plasticity and memory along with down regulation of plasticity genes in compared to males. A higher Aβ pathology and neuroinflammatory response is seen in female AD mice compared to male AD (represented as blue).

Collectively, our study confirms that APP/PS1 female mice show a stronger inflammatory response and a downregulation of gene expression associated with plasticity factors that may contribute toward the impairments of various forms of hippocampal Schaffer collateral LTP and formation of associative memories (Figure 1). More work remains to be done to better understand how sex differences in the brain intersect with risk factors to influence synaptic function not only in demented brains, but also during the normal aging process. Our work, along with others in this field, also emphasizes the importance of including biological sex as variable in many research settings, particularly studies exploring aging [7] and how they impact different disease states [8]. In the long run, the underrepresentation of female biology in biomedical research will hamper the development of effective drugs with negative consequences on women’s health.
